# Long-Term and Transgenerational Effects of Stress Experienced during Different Life Phases in Chickens (*Gallus gallus*)

**DOI:** 10.1371/journal.pone.0153879

**Published:** 2016-04-22

**Authors:** Maria Ericsson, Rie Henriksen, Johan Bélteky, Ann-Sofie Sundman, Kiseko Shionoya, Per Jensen

**Affiliations:** 1 AVIAN Behavioural Physiology and Genomics Group, IFM Biology, Linköping University, Linköping, Sweden; 2 Department of Clinical and Experimental Medicine, IKE, Division of Cell Biology, Linköping University, Linköping, Sweden; Radboud University Nijmegen, NETHERLANDS

## Abstract

Stress in animals causes not only immediate reactions, but may affect their biology for long periods, even across generations. Particular interest has been paid to perinatal stress, but also adolescence has been shown to be a sensitive period in mammals. So far, no systematic study has been performed of the relative importance of stress encountered during different life phases. In this study, groups of chickens were exposed to a six-day period of repeated stress during three different life phases: early (two weeks), early puberty (eight weeks) and late puberty (17 weeks), and the effects were compared to an unstressed control group. The short-term effects were assessed by behaviour, and the long-term and transgenerational effects were determined by effects on behavior and corticosterone secretion, as well as on hypothalamic gene expression. Short-term effects were strongest in the two week group and the eight week group, whereas long-term and transgenerational effects were detected in all three stress groups. However, stress at different ages affected different aspects of the biology of the chickens, and it was not possible to determine a particularly sensitive life phase. The results show that stress during puberty appears to be at least equally critical as the previously studied early life phase. These findings may have important implications for animal welfare in egg production, since laying hens are often exposed to stress during the three periods pinpointed here.

## Introduction

During the perinatal period vertebrates are particularly sensitive to stress, due to the speed and complexity of brain development during this time (reviewed by [1]). Glucocorticoid exposure to the maturing brain induces alterations in gene expression, and causes a hyper-responsive HPA-axis and increased anxiety behaviour [2–6]. Extensive literature suggests that stress during early life stages can induce persisting effects on physiology [7–9], behavior [7, 9–12] and immune function [2]. For reviews, see for example [13] or [1]. However, age at the time of stressor exposure, as well as duration and type of the stressor can affect the outcome [7, 12, 14], and the timing of the stress appears important for the long-term consequences [15, 16].

Adolescence, the period of maturation of social and cognitive behaviour [17], has been suggested to be highly sensitive to stress in rodents [18] due to the considerable behavioral, morphological and hormonal changes that occur during this period. As reviewed by [19], adolescent rats have a prolonged glucocorticoid response to aversive stimuli compared to adults exposed to the identical stressor, and stress during adolescence can cause behavioural changes indicative of increased anxiety in adulthood and affect sexual behaviour [20, 21]. In birds, this period is more rather referred to as puberty [22–24]

In altricial birds, unpredictable food supply during the juvenile stage can affect a wide range of phenotypic traits [25] which may persist into adulthood [26]. In precocial birds, early postnatal stressor exposure is not well-investigated but it has been demonstrated that 2 week old Japanese Quail displayed increased behavioural flexibility compared to controls as a consequence of stressor exposure [10]. In our model species, the chicken (*Gallus gallus*), early environmental deficiencies (for example lack of perches, lack of maternal care, unsuitable flooring material) can cause development of abnormal behaviors such as severe feather pecking and cannibalism [27–29]. In birds, however, little is known regarding stress sensitivity during puberty [22]. Several systems are still developing in the chick post hatch, including synapse formation in the brain until 10 weeks of age [30]. The period of puberty seemingly occur at different ages in male and female chickens. In males a definite comb is visible at 8 weeks of age [31], and at this period a progressive rise of circulating testosterone until week 16 was observed [32]. For domestic chicken females, onset of egg laying is at 19–20 weeks of age [33], indicating the final maturation of the reproductive system. Hence, we wanted to make a broad investigation of the effects of stress when encountered during the above mentioned potentially sensitive periods.

Phenotypic effects of stress may persist throughout life, but also across generations, as shown in rodents as well as in chickens [34–36]. Such transgenerational effects are related to modified gene expression profiles in hypothalamus, a part of the brain closely involved in control of the HPA-axis activity [35, 37], indicating epigenetic regulation.

The aim of the present experiment was to search for stress sensitive periods inducing long-lasting and transgenerational effects on behaviour, physiology and gene expression in chickens. We exposed chickens to similar stressors at three different developmental periods, and hypothesized that this would induce different alterations in behaviour, HPA-axis sensitivity and gene expression, both within and across generations.

## Methods

### Ethical note

This study was approved by Linköping Council for Ethical Licensing of Animal Experiments, ethical permit no 122–10.

### Animals

Newly hatched Hy-Line White Leghorn chicks (97 females and 103 males) were obtained day-old from a local hatchery (Swedfarm, Linghem, Sweden), individually marked with numbered wing-clips, and housed in the same room in 4 identical pens (0.75 x 1.5 x1.8 m; L x W x H) equipped with heat lamps. At 3 weeks of age, all chicks were placed together in one single pen (1.5 x 3.0 m) equipped with perches. At 5 weeks of age, the chicks were moved to the rearing pens, where they were kept for the rest of their lives. Here they were separated by sex and housed in two neighboring, identical multi-level system pens measuring 3.0 x 2.5 x 3.0 m (L x W x H) with perches and nests. During all housing conditions, the floor was covered with wood chips, and the chickens were kept on a 12:12 hour light:dark schedule with access to conventional chicken feed and water *ad libitum*.

After the finishing of all testing, the birds were paired to generate an offspring generation, as outlined below. They were culled when 247 days old, and brain samples were obtained as described below.

### Treatment groups

At 1 day of age the chicks were divided into four approximately equally sized groups. A control group (C) was not exposed to any experimental stress at any age (24 females and 25 males). The three remaining groups were each assigned a stress exposure age: as juvenile at 2 weeks of age (2Wstress; 25 females and 25 males); at 8 weeks (8Wstress), representing early puberty (24 females and 26 males); and at 17 weeks of age (17Wstress), representing late puberty (24 females and 25 males). Each group was only exposed to the stress treatment at the designated age, and for the rest of their lives, they were treated exactly as the C-group.

As described above, all chickens, regardless of treatment were kept in the same pen from three weeks of age, then from five weeks in two pens with treatment groups equally divided between the pens. They were housed in these pens at all times except during the stress treatment periods, at which time the birds exposed were moved to another room and exposed to the stress treatments (as described below), before being moved back to the same home pens. Since birds from all treatment groups were kept together, the risk of group confoundment was minimized, and if anything, any differences caused by the different treatments would tend to be decreased by this procedure.

### Stress treatment

At their designated week (2, 8 or 17 weeks), the birds were exposed to three different stressors applied during six consecutive days. On day one and four, the stressor was food frustration (8 x 5 min per day of exposure to unreachable meal worms; applied to the whole group simultaneously), on day two and five, the stressor was 5 x 5 min of physical restraint in a cloth bag (applied to each individual separately), and on day three and six the stressor was social isolation in a card-board box four times per day (again applied to each individual separately; 30–60 min per instance). The stressors were applied at random times between 0800 and 1700 h. These treatments have previously been found to induce a reliable increase in HPA-axis activity at all ages [38], and the fact that all stressors were applied repeatedly at all ages should ascertain a similar impact on the chickens stressed at different ages.

### Growth

All chickens were weighed on the day of hatch and at day six, 146 and 247. The chickens were also weighed the day before and after their respective stress treatment. The control group was weighed at the same instances.

### Short term behavioural effects

The short-term effects of the treatment were assessed by comparing the behavior of the stress treated birds to that of the control birds directly following the final day of each stress period. The undisturbed behavior was assessed by placing two chicks of the same sex and treatment in a separate pen (2Wstress: 75 x 75 x 180 cm, 8Wstress and 17Wstress: 100 x 100 x 210 cm) with ad libitum access to feed and water, and recording their behavior for 25 min after a 30 min habituation period. In total 32 birds were included in this part for the 2Wstress and 8Wstress, groups and for the 17Wstress, 28 birds were included. At each age, the same number of control birds was tested simultaneously. Behaviour was recorded by instantaneous sampling every 60 s according to the ethogram provided in S1 Table. Immediately after the end of the recordings, lights were turned off and an unfamiliar object (a fishing float, a red soda can or an orange cardboard box) was placed in the food tray, where after light was turned on and for 5 minutes the behaviour recordings continued as before.

During the week following the end of treatment, a tonic immobility test (TI; a measure of fearfulness) was conducted on all birds as described previously [38]. The duration of tonic immobility, i.e. the times until first movement and until righting was recorded with an upper limit set to 10 min.

### Long-term behavioural effects

The long-term effects were assessed in all birds by two behaviour tests commencing when the birds were 211 days old, i.e., about ten weeks after the end of the last stress treatment.

A subgroup was tested in the emergence test (n = 77) at the age of 212 ± 1 day. The test was a modified version of the light-dark emergence test, commonly used in rodents to assess anxiety and exploratory behaviour [39]. The birds were placed individually in a closed cardboard box measuring 70 x 27 x 29 cm. The box had a guillotine-type door that was raised after 2 minutes of acclimatization. The latency until the head emerged (HE) from the box was recorded, as well as until the full body (FE) had emerged from the box. If the bird had not emerged from the box after 5 min, it was given a maximum score.

To assess the ability to recover from an acute stress event, the stress recovery test was performed, commencing on 217 days of age, described in detail by [40]. Briefly, the behaviour of individual birds was recorded 30 min after 3 min of physical restraint and compared to the undisturbed behaviour 30 min before the restraint. The test was combined with a novel object test; a red and white aluminum can was placed in the arena following the 30 min recovery period, and the behaviour was recorded during an additional 10 min. For the complete ethogram see S2 Table.

### HPA-axis sensitivity

At 29 weeks of age, the reactivity of the HPA-axis was assessed by quantifying the corticosterone (CORT) response to restraint [41]. Birds were blood sampled from the wing veins and baseline samples were obtained within 3 min after the person entered their home pen. Immediately after this, each bird was placed in a large-meshed net hanging above the floor. The birds were blood sampled again at 10 min and 30 min after being placed into the bag and returned to the aviaries after the last sampling. Blood was collected with heparin-coated capillaries into eppendorf tubes, which were then centrifuged and the plasma was stored at −20°C until further analysis (see below).

### Effects on offspring

To examine possible transgenerational effects, all hens were paired with males from the same treatment groups at five months of age, and eggs were incubated and hatched as previously described [35]. After wing-marking, all offspring (F1) were raised in a single group. They were weighed at hatching and at 11, 28 and 56 days. At seven weeks the offspring were culled and brain samples were obtained for gene expression analysis as described below.

At 11 days of age the F1-birds were tested in an open field test, as described previously [42]. Activity in the open field arena was recorded by automatic video tracking, using the EthoVision software (Noldus Information Technology, Wageningen, the Netherlands). We assessed latency to first movement, total time spent in the middle of the arena, total time spent close to the sides in the arena and total distance moved during the 5 min testing.

At 18 days of age all F1-birds were tested in a tonic immobility test, following the same procedure as described above for the parents. At the age of 23 or 24 days the offspring (C (n = 20), 2Wstress (n = 16), 8Wstress (n = 13), 17Wstress (n = 16)) underwent an identical emergence test as described for the parents. When seven weeks old, HPA-axis reactivity was determined by the same CORT-sampling procedure as used for the parents (see above), with the exception that the birds were placed on the floor in smaller nets.

### Hormone assay

The concentrations of CORT in the plasma samples were determined using a commercial CORT enzyme-linked immunosorbent assay (ELISA) kit (Enzo Life Sciences, NY, USA). All samples were tested in duplicate following a standard protocol (see online manual: http://www.enzolifesciences.com/ADI-900-097/corticosterone-eia-kit/). The CORT assay had a sensitivity of 26.99 pg/ml and inter-assay and intra-assay coefficients of variation were 15.40% and 6.37% respectively.

### Gene expression

After culling, the birds were decapitated and their brains were removed, dissected and frozen in liquid nitrogen within 15 min. From the brains, we dissected the ventral half of the mid-brain, mainly consisting of thalamus and hypothalamus [35]. Samples from parents and offspring were treated and analysed with the same methods, according to the following procedure:

Brain samples were homogenized with 1 ml TRI-reagents (Ambion) in Lyzing matrix D tubes containing ceramic beads (MP Biomedicals) using a FastPrep® -24 homogenization system. The rest of the extraction was performed according to the TRI-reagent manufacturer’s protocol, with the modification that 0.25 ml isopropanol and 0.25 ml RNA-precipitation solution (1.2 M NaCl, 0.8 M disodium citrate) was added to the samples during extraction. RNA quantity and quality was measured using a NanoDrop® ND-2000c (Thermo Scientific) and a Bioanalyzer® instrument (Agilent Technologies). All samples were individually treated with DNase I (Thermo Scientific). Then first-strand cDNA was synthesized using Maxima H Minus First Strand cDNA Synthesis Kit (Thermo Scientific), followed by second strand cDNA synthesis, phenol/chloroform extraction and precipitation. Samples were pooled (see below), labeled and hybridized to NimbleGen 12 x 135k custom gene expression arrays (Roche NimbleGen) and scanned using a NimbleGen hybridization station and scanner. For gene expression data, the dscDNA samples from the brains were pooled in groups of three within generation, treatment and sex before labeling and hybridization. In this way, we had one microarray with a pool of three individuals for each generation, sex and treatment group, altogether 48 birds. The microarray data can be accessed at Annotare (http://www.ebi.ac.uk/arrayexpress/) under accession E-MTAB-3623.

### Statistics

Statistical analyses were performed in SPSS version 22 or Statistica 12 (StatSoft, Tulsa, OK, USA), and in R (gene expression). One-way ANOVA was used to determine between group differences in egg mass laid by stress treated females. The undisturbed behaviour was analyzed using a factorial ANOVA with sex and treatment in the model. Open field behavioural data for offspring and body mass data for all treatment groups and their offspring were analysed by two-way ANOVA with treatment and sex as variables. Plasma CORT values from the restraint test were analysed using a mixed repeated-measure ANOVA with treatment and sex as between-subject factors and time as within-subject factor. A repeated measures ANOVA was used for analyzing the stress recovery/novel object test. The between treatment group difference in duration of tonic immobility and the emergence test were examined by using Kaplan-Meier survival analysis, with a post hoc χ^2^-test or Cox’s F-test. The statistical significance level was set at P < 0.05. If 0.05 > P < 0.1, the results were considered a tendency and a post-hoc analysis was performed.

Normalization of the arrays was done with the RMA method using the DEVA software (NimbleGen). Gene expression was analyzed using R/Bioconductor (www.bioconductor.org).

For each stress treatment group, the expression was compared to the controls. The genes were listed according to fold-change, disregarding P-values, and the top 1000 genes on these lists were obtained for each of the stress groups. To analyse pathways affected by the stress treatment, we selected genes overlapping between all treatment groups (i.e. found on the top 1000 list of all three groups) and subjected those to gene ontology analysis. For this purpose, Ensembl gene IDs were matched to the chicken array using the Manteia web tool (manteia.igbmc.fr).

To analyse possible transgenerational effects of the stress treatments, we generated the same top 1000 fold-change lists for the F1-birds, comparing expression in the offspring of each treatment group to offspring of the controls. We then selected those genes, which appeared on the top lists of both the parents and the offspring in each treatment group, and calculated the correlation coefficients for each comparison. This analysis shows the extent to which altered gene expression in parents stressed at a certain age is mirrored in the expression profiles of their offspring.

## Results

### Short-term effects

**Fig 1:**
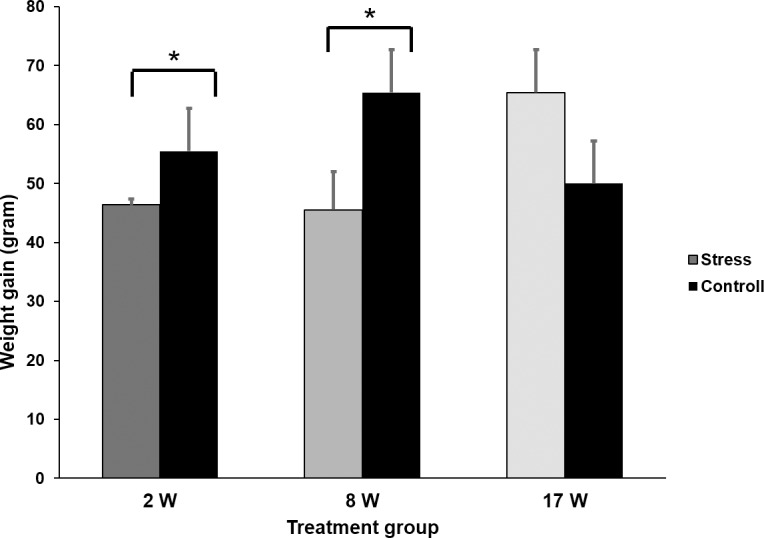
Weight gain parental generation. Weight gain during the week of stressor exposure for each treatment group, compared to its respective control group. * = P < 0.05.

Short-term effects were assessed immediately after the completion of each stress period, so when the chickens aged two (2Wstress), eight (8Wstress) and 17 weeks (17Wstress) respectively. Hence, this applies to all tests described in this section. Compared to C, 2Wstress and 8Wstress birds gained significantly less weight during the stress week. (2Wstress: F_1, 93_ = 68.5; P < 0.0001; 8Wstress: F_1, 77_ = 12.18; P = 0.0008), whereas there were no significant effects on weight gain in the 17Wstress group (Fig 1).

With respect to undisturbed behaviour, 8Wstress birds showed significantly less alert walking (F_1, 28_ = 4.842; P = 0.036) compared to C and tended to perform more comfort behaviour (F_1, 28_ = 3.678; P = 0.065), while no significant differences were seen for 2Wstress and 17Wstress. In the novel object situation, the 8Wstress group performed significantly less walking alert than C (F_1, 28_ = 4.84; P = 0.036) and tended to perform more comfort behaviour (F_1, 28_ = 3.68; P = 0.065), while again, there were no significant effects for either 2Wstress or 17Wstress. No other behaviours were significantly different between the stress and control groups.

In the TI-test (Table 1), a significantly shorter latency both to first movement and to righting was observed in 2Wstress and 8Wstress compared to C (Table 1). The 17Wstress group tended to have a longer latency to first movement and a shorter time to righting compared to C.

**Table 1:** Latencies (s) to First movement and Righting in the Tonic Immobility test applied immediately after the end of each stress period. For each stress group (2Wstress: stressed at two weeks of age; 8Wstress: stressed at eight weeks; 17Wstress: stressed at 17 weeks) data are shown in comparison to the non-stressed control chickens tested at the same time. P-values show the significance levels of the Cox’s F-values obtained from survival tests.

Treatment	First movement			Righting		
	Stressed	Control	P	Stressed	Control	P
2Wstress	74 ± 81	145 ± 168	0.011	203 ± 171	316 ± 224	0.009
8Wstress	137 ± 99	200 ± 126	0.013	285 ± 166	376 ± 182	0.022
17Wstress	190 ± 171	134 ± 94	0.058	339 ± 198	419 ± 209	0.062

### Long-term effects

Long-term effects were assessed ten weeks after the end of the last stress period, when the birds were 190 days old by means of two different behavioural tests. This therefore applies to all results in this section.

**Fig 2:**
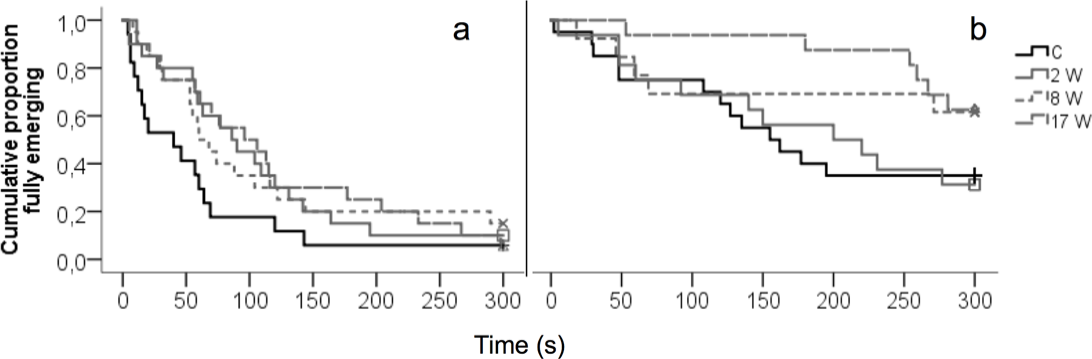
Emergence test. Latency until head and full emergence for (a) the birds in the parental (experimentally treated) generation and (b) their offspring.

In the emergence test, birds from all stressed groups tended to emerge earlier than the controls, both for head emergence (χ^2^ = 7.1, df = 3, P = 0.07) and full emergence (χ^2^ = 7.1; df = 3; P = 0.07) (Fig 2A).

**Fig 3:**
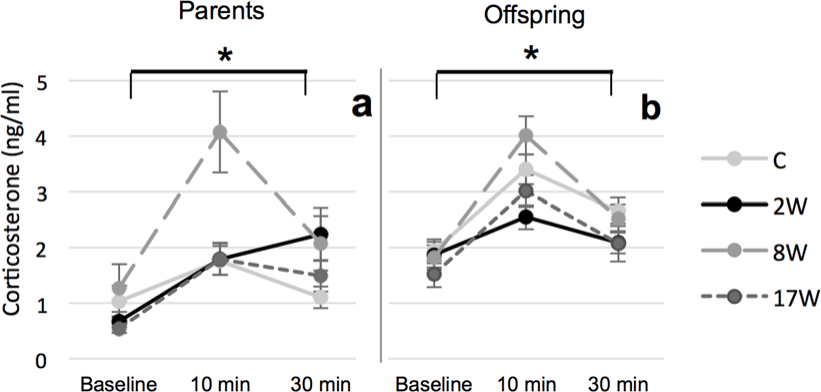
HPA-axis reactivity. Corticosterone levels for the different treatment groups and the control group at baseline levels, and 10 and 30 minutes after onset of physical restraint. (a) parekantal generation, (b) offspring. * = P < 0.05.

There was a significant effect of treatment on the adult HPA-axis response (F_6, 76_ = 3.147, P(treatment*time) = 0.008) (Fig 3A). While baseline plasma CORT levels were similar between groups (F_3, 42_ = 1.630; P = 0.197), they differed significantly after 10 min restraint (F_3,42_ = 5.258; P = 0.004). Post hoc tests revealed that birds in the 8Wstress group had significantly higher CORT levels after 10 min than any of the other treatment groups including control birds (8Wstress vs C: P = 0.009; 8Wstress vs 2Wstress, P = 0.012; 8Wstress vs 17Wstress, P = 0.010). At the end of the 30 min restraint stress there were no significant differences between the groups (F_3,42_ = 1.089; P = 0.364).

In the stress recovery test, there were no significant differences in baseline behaviour between the groups. During the recovery period, there was a tendency for a Time*Treatment effect on the frequency of “Escape” (F_6, 78_ = 2.01; P = 0.075), where the 8Wstress group tended to show more escape attempts. During the novel object presentation in the recovery period, there was again a tendency for an effect of treatment on “Escape” (F_3, 39_ = 2.57; P = 0.068), where 8Wstress again tended to have more escape attempts. In the TI-test in the adults, no significant differences were observed between the groups in either time to first movement or time to righting.

**Fig 4:**
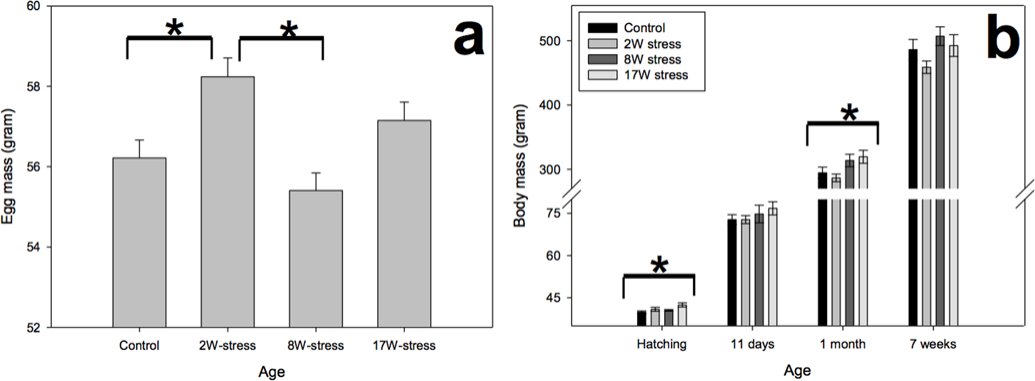
Offspring egg mass and growth. Weight of eggs laid by the parental females (a). Weight of the offspring at four different ages (b). * = P < 0.05.

Stress treatment had a significant effect on egg mass (F 3,318 = 6.658; P < 0.001), and post-hoc test revealed that 2Wstress females laid significantly heavier eggs than 8Wstress (P = 0.001) and C females (P = 0.016) (Fig 4A).

### Effects on offspring

At hatch, there was a significant effect of parental treatment on offspring weight (F_3, 66_ = 3.204; P = 0.029), but post-hoc analysis showed that only the offspring of 17Wstress birds were significantly heavier than offspring of controls (P = 0.018). No difference in body mass was observed at 11 days (F_3, 66_ = 1.197; P = 0.318), while at one month of age there was again a significant effect (F_3, 66_ = 2.872; P = 0.043), where offspring of the 8Wstress group were significantly heavier than offspring of 2Wstress (P = 0.025), and at seven weeks of age there was a tendency for a treatment effect (F_3,59_ = 2.666; P = 0.056) (Fig 4B).

There was no significant effect of parental treatment on duration of TI (Survival analysis: χ^2^ = 5.365; P = 0.15). In the open field test, there was a tendency for an effect of parental treatment on latency to start moving (Mann-Whitney U-test, P = 0.094), with 8Wstress and 17Wstress offspring having a shorter latency (1.1 ± 0.6 s. and 1.5 ±1.1 s. respectively) than control and 2Wstress offspring (9.2 ± 4.6 s. and 8.7 ±4.5 s. respectively) but no effects of treatment on distance moved (F_3_, _62_ = 1.798, P = 0.157). There was a tendency (F_3_, _62_ = 2.365, P = 0.080) for 8Wstress offspring to spend less time at edges of the open field arena (77.5 ± 12.6 s.) than the other offspring groups (C: 129.6 ± 12.6 s., 2Wstress: 103.9 ± 17.4 sec., 17Wstress: 134.8 ± 20.5 s.). In the emergence test, a tendency for a parental treatment effect was seen for full emergence (χ^2^ = 7.3; df = 3; P = 0.06) (Fig 3B). A post-hoc pairwise comparison (Mann Whitney U-test) showed a significantly longer latency until emerging from the box in 17Wstress offspring compared to C (P = 0.02).

There was a significant effect of parental treatment on HPA-axis response (F_6, 112_ = 2.773, P(treatment*time) = 0.041) (Fig 3B). Whereas there were no significant differences in basal plasma CORT levels (F_3, 56_ = 0.646, P = 0.439) or CORT levels at the end of the 30 min restraint (F_3, 56_ = 1.293, P = 0.328), there was a significant effect of parental treatment on cort levels after 10 min restraint (F_3, 56_ = 4.301, P = 0.009), with offspring of 8Wstress having significantly higher plasma CORT levels than offspring of 2Wstress (P = 0.011).

### Gene expression

Out of the top 1000 differentially expressed genes in each treatment group, there were 98 overlapping between all treatments. A complete list of the 98 genes overlapping on the top lists can be found in S3 Table.

**Fig 5:**
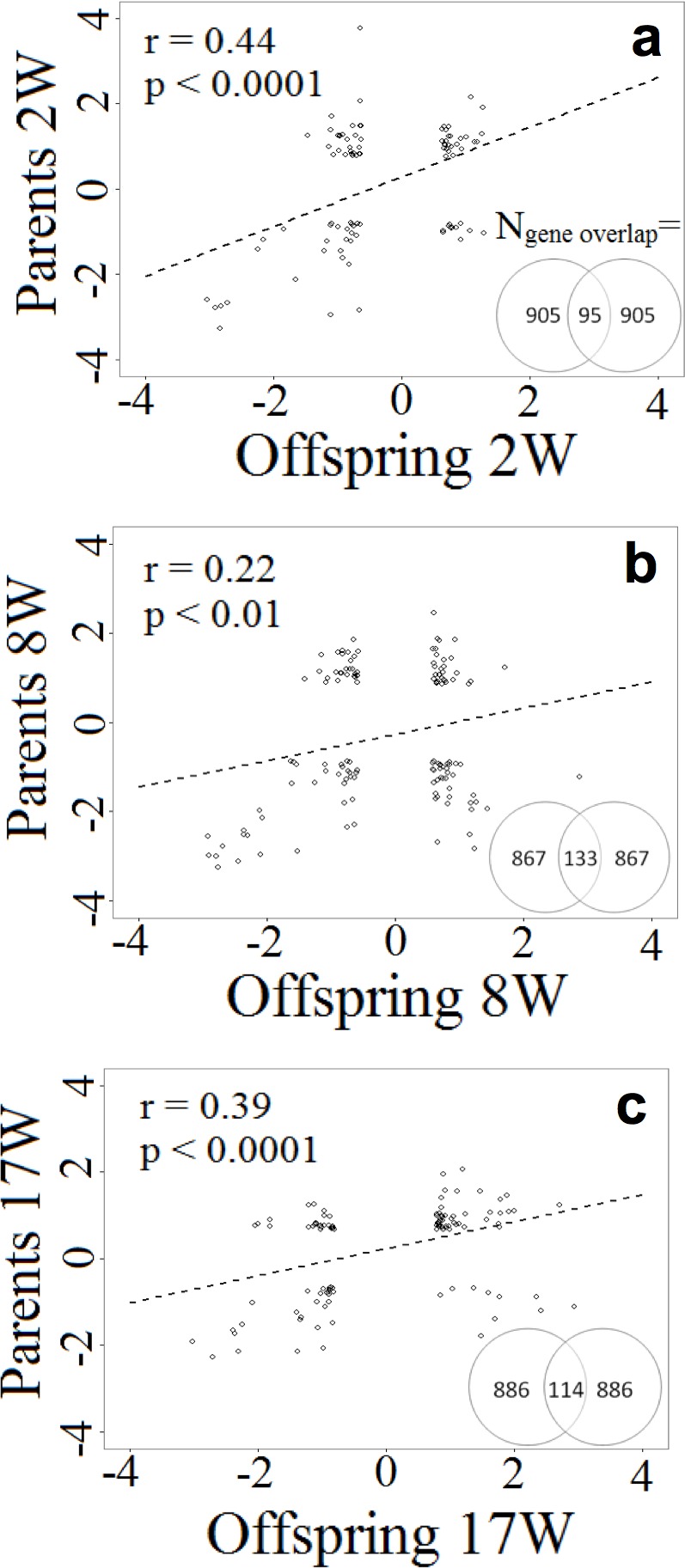
Gene expression analysis. Transgenerational correlation of differential gene expression (DE; log2 fold change) of the overlapping top DE genes within treatment groups. The graphs show the correlation between the difference in expression comparing each stressed group to the controls. a) Parents stressed at two weeks, b) parents stressed at eight weeks and c) parents stressed at 17Wstress. The Venn-diagrams indicate the number of overlapping genes, i.e., those on which the correlation analyses are based, within each treatment.

With respect to transgenerational effects, the list of the 1000 most differentially expressed genes in each treatment group and in each generation contained an overlap of between 95–133 genes over generations. This represents genes which showed a high fold change in both the stressed parents and the offspring from the same stress group. The correlation between the fold changes of the overlapping genes was significant for all three treatment groups, but was strongest in the 2Wstress and 17Wstress groups (Fig 5).

A Gene Ontology (GO) analysis of the genes with the strongest expression difference in all treatment groups (i.e., gene expression effects in the animals exposed to the stress compared to the controls) revealed enrichment of terms with a strong connection to stress. The significant terms were “extracellular region part” (P < 0.05) with 14% of genes, amongst those *corticotropin releasing hormone binding protein (CRHBP)* and *oxytocin (OXT)*, “sensory perception” (P < 0.05), and the almost significant term “neurological system process” (P = 0.052). Among other potentially interesting genes among the overlapping ones, we found, for example, *progesterone receptor (PGR)* and *glycoprotein hormones*, *alpha subunit (CGA)*.

Focusing on the transgenerationally overlapping DE genes (i.e., genes differentially expressed both in the stressed animals and in their offspring) showed enrichment of a few GO terms (p < 0.05) of relevance to the stress treatment, such as “behaviour” (10 of 62 genes) and “regulation of synaptic transcription” (6 out of 62) in 2Wstress animals and their offspring, “sequence-specific DNA binding” (11 out of 94) in 8Wstress and their offspring, and “serotonin transmembrane transporter activity” (2 out of 80) and “system process” (14 out of 80) in 17Wstress and their offspring. Among the genes affected, the *solute carrier family 6 (neurotransmitter transporter)*, *member 4 (SLC6A4)* showed a decreased expression in both parents and offspring in all stress groups. Other genes with a modified expression in both generations were *dopamine beta-monooxygenase*, ***(****DBH)* in 2Wstress and 8Wstress and *oxytocin (OXT)* in 2Wstress and 17W. In 2Wstress only, there was an effect in both generations on the expression of *Prothyroliberin Thyroliberin Thyroliberin-like (TRH)* and *tryptophan 5-hydroxylase 2 (TPH2)*, and in 8Wstress on *Arginine vasopressin receptor 2 (AVPR2)*. In 17Wstress only, there was a a fold change effect on *D(1A) dopamine receptor (DRD1)* and *solute carrier family 18 (vesicular monoamine)*, *member 2 (SLC18A2)* in both generations.

A complete list of all genes overlapping across generations is found in S4 Table.

## Discussion

Our results show that the short-term effects of stress were more pronounced in young and early pubertal chickens, with weaker effects in late puberty. However, long-term effects, lasting beyond sexual maturity, were most pronounced in birds which had been stressed during early puberty. While gene expression profiles were modified in the offspring of all treatment groups, behavioural and physiological transgenerational effects were mainly observed for birds stressed in late puberty. Taken together, the results show that the effects of stress in chickens varied both in magnitude and duration depending on when the stress was experienced.

The short-term effects observed on weight gain in the two youngest groups were similar to the results obtained in a study on Japanese Quail chicks [10]. The lack of such effects in the 17Wstress group could indicate that the stress treatment affected the older birds less in the short-term, which is supported by the behavioural data. Our results therefore suggest that stress in chickens has conceivable short-term effects mainly if experienced during early life or early puberty. The recordings of undisturbed behaviour revealed very few and rather weak effects in the 8Wstress group only. Birds stressed at eight weeks performed less walking alert, and reduced activity is a commonly observed result of previous stress in chickens [10]. However, the fact that so few of the recorded behaviours showed an effect at any of the treatments means that the results should be interpreted with great care.

Considering long-term effects, only birds stressed in early puberty showed higher CORT response after restraint. This shows that stressful experiences in early puberty can induce long-term biological effects. Hence, early puberty appears to be a sensitive period during development in chickens, indicating that the neural and endocrine systems during this period respond and change to current environmental inputs.

Exposure to stress prior to sexual maturity has been shown to have profound effects on the development of the HPA-axis in birds [43–45], but to our knowledge, no previous studies have presented a cohesive picture of the long-term effects of stress during different stages of puberty. In rodents, however, adolescent stress has been shown to affect the HPA-axis, although the direction of the effects differs between studies, possibly due to differences in stressors and intensity [46].

Pronounced stress-induced alterations in HPA-axis activity during the pubertal period has been reported in several bird species (for review see [47]), supporting that puberty may be a particularly sensitive time for the development of the HPA-axis. Often these neuroendocrine changes are linked to altered behavioural responses. In the present experiment, several tendencies for behavioural changes were seen in the adult chickens as a consequence of the early or pubertal stress exposure. Taken together, the behavioural data indicate that stress at eight weeks increased the general level of fearfulness (as measured by emergence, TI and stress recovery) more than when stress was induced at any of the other ages.

There were several significant transgenerational effects of stress on behavior as well as on HPA-axis activity. However, the results did not pinpoint any particularly sensitive period, even if 8Wstress stress had the strongest effect on the CORT response in the offspring. Previously, it has been shown that stress during the first four weeks of life can induce transgenerational effects on the HPA-axis in chickens [37]. More research is needed to elucidate the precise life phases during which the HPA-axis is amenable to transgenerational modifications.

Possible mechanisms whereby stress can cause transgenerational effects include heritable epigenetic modifications, which can affect brain gene expression [35]. The significant correlations of differential expression across generations for all three treatments support that heritable epigenetic modifications may have been induced by the stress treatments. Since the effects were strongest in the 2Wstress and 17Wstress groups, this indicates that those periods are particularly susceptible to transgenerational modifications of brain gene expression. The results corroborate those previously reported [37] for birds stressed during the first four weeks of life, and those of reference [35], and suggest that transgenerational responses may be induced by stress at different life phases.

Apart from the increased basic understanding of the development of stress sensitivity in birds, our results also have important practical implications for chicken welfare. Chicks are exposed to many different stressors both during the early life phases (for example, sex sorting and transport) and puberty (for example, transport and regrouping) [48]. Such routines may obviously affect chickens for long periods and could potentially be important for the welfare of millions of laying hens. For example, de Haas et al [49] found that chickens in more stressed parent flocks produced offspring with higher levels of severe feather pecking. Interestingly, in their study, feather pecking peaked at around five-six weeks, close to the sensitive of eight weeks identified by us. Hence, although further applied research is needed, our results may have important implications for husbandry routines in egg production.

In conclusion, we have shown that stress may induce lasting and transgenerational modifications of behaviour, HPA-reactivity, and hypothalamic gene expression when experienced at different phases of life. The effects varied dependent on age at stress, and we can therefore not pinpoint any of the three periods studied as particularly sensitive. However, our results show for the first time that puberty in chickens appears to be at least equally critical as the previously studied early life phase.

## Supporting Information

S1 Table Ethogram for undisturbed behaviour and novel object test. (DOCX)

S2 Table Ethogram used for the stress recovery/novel object test. (DOCX)

S3 Table Differentially expressed (DE) genes found among the top 1000 DE genes from each treatment in the parental generation. (DOCX)

S4 Table Differentially expressed probes for each treatment group when comparing parents with their offspring. (DOCX)

S1 Data Raw data for behavioural tests, corticosterone values and weights. (XLSX)
