# Supplementary material for: Long-Term and Transgenerational Effects of Stress Experienced during Different Life Phases in Chickens (Gallus gallus)
Source: PLoS One. 2016 Apr 22;11(4):e0153879. doi: 10.1371/journal.pone.0153879 (PMC4841578; doi:10.1371/journal.pone.0153879)
Supplement: S1 Table — (DOCX) [file pone.0153879.s001.docx]

**S1 Table.** **Ethogram for undisturbed behaviour and novel object test.** Behaviours were pooled into the variables prior to analysis.

| **Variable** | **Behaviour** | **Description** |
| --- | --- | --- |
| **Foraging** | Feed | Eating from food tray. |
|  | Drink | Drinking from water bell |
|  | Explore ground | Walking or standing with head close to ground (below back) or pecking on ground, eyes focusing on ground items, or scratching on ground with feet |
|  | Explore object | Head close to object of interest or pecking at/manipulating object, eyes focusing on object |
| **Passive** | Stand/sit | Standing (legs erect) or sitting (legs bent) on ground or perch; may be relaxed or alert |
| **Walk alert** | Walk alert | Moving and attending to the surrounding |
| **Walk** | Walk | Moving with reduced attention; neck short, no alert head movements |
| **Escape** | Escape | Attempt to escape out from the test arena by jumping or making fly attempts towards the roof/walls |
| **Comfort behaviour** | Preen | Uses beak to trim and arrange feathers |
|  | Stretch wing | Stretches wing straight backwards |
|  | Stretch leg | Stretches leg |
|  | Yawn | Gape |
|  | Feather ruffle | Erects feathers, ruffles, and shakes body |
|  | Wing flap | Flaps wings while standing on ground or perch |
| **Social behaviour** | Social | Social, non-aggressive, peck on cage mate |
| **Crow** | Crow | Cockerel crowing |
| **Explore novel object** | Explore novel object | Eye/s focusing on object, with attentive body posture moving toward object; or head close to novel object with eyes focusing on object; or pecks at novel object; or uses beak to manipulate object |
| **Freeze** | Freeze | Stiff posture, stand, sit or lie motionless, vigilant, open eyes |
