# Supplementary material for: Long-Term and Transgenerational Effects of Stress Experienced during Different Life Phases in Chickens (Gallus gallus)
Source: PLoS One. 2016 Apr 22;11(4):e0153879. doi: 10.1371/journal.pone.0153879 (PMC4841578; doi:10.1371/journal.pone.0153879)
Supplement: S2 Table — (DOCX) [file pone.0153879.s002.docx]

**S2 Table. Ethogram used for the stress recovery/novel object test.**

| Behaviour | Definition |
| --- | --- |
| Feed | Feeding from food container |
| Drink | Dipping beak into water bell |
| Foraging | Focus on the floor, pecking and scratching the ground |
| Alert behaviour | Stands, sits or walks with open eyes, attending to the surrounding |
| Relaxed behaviour | Stands or sits with reduced attention, eyes may be partly closed, neck short, no alert head movements |
| Freeze | Stiff posture, stand, sit or lie motionless, vigilant, open eyes |
| Escape | Attempt to escape out from the test arena by jumping or making fly attempts towards the roof. |
| Comfort behaviour | Dust bathing, preening, scratching or feather ruffles |
| Crowing | Male crowing |
| Other | Other behaviours not defined in the ethogram |
| Explore object | From near or distance, eyes clearly focused on the object |
| Peck object | Distinct bovement of beak towards object, beak touches object |
| Manipulate object | Using beak or feet to move object |
