# Supplementary material for: Long-Term and Transgenerational Effects of Stress Experienced during Different Life Phases in Chickens (Gallus gallus)
Source: PLoS One. 2016 Apr 22;11(4):e0153879. doi: 10.1371/journal.pone.0153879 (PMC4841578; doi:10.1371/journal.pone.0153879)
Supplement: S3 Table — (DOCX) [file pone.0153879.s003.docx]

**S3 Table.** **Differentially expressed (DE) genes found among the top 1000 DE genes from each treatment in the parental generation.** Probe transcript IDs are given for all 98 probes, along with Ensembl Gene IDs and gene annotations when available. Location and start site for the probes are provided as well.

| Probe ID | Ensembl Gene ID | Gene | Chromosome | Start (bp) |
| --- | --- | --- | --- | --- |
| ENSGALT00000013512 | ENSGALG00000008298 | LRRC17 | 1 | 13834965 |
| NM_001031332 | ENSGALG00000004897 | GTSE1 | 1 | 16895123 |
| NM_001001301 | ENSGALG00000010198 | TPH2 | 1 | 38382634 |
| ENSGALT00000018392 | ENSGALG00000011271 | LUM | 1 | 45880845 |
| ENSGALT00000026770 | ENSGALG00000016584 | EGFL6 | 1 | 126473646 |
| 603601429F1 | - | - | 1 | 158951976 |
| ENSGALT00000023466 | ENSGALG00000014545 | LOC427010 | 1 | 165980409 |
| NM_001030541 | ENSGALG00000017046 | POSTN | 1 | 176288240 |
| ENSGALT00000036466 | ENSGALG00000017046 | POSTN | 1 | 176288241 |
| NM_205262 | ENSGALG00000017195 | PGR | 1 | 187457390 |
| ENSGALT00000038721 | ENSGALG00000023430 | EN2 | 2 | 7817560 |
| NM_205268 | ENSGALG00000016112 | NOV | 2 | 142002873 |
| ENSGALT00000025959 | ENSGALG00000016112 | NOV | 2 | 142002898 |
| ENSGALT00000028816 | ENSGALG00000018109 | - | 3 | 25325903 |
| 603867870F1 | - | - | 3 | 56444558 |
| ENSGALT00000037452 | - | EYA4 | 3 | 58370070 |
| ENSGALT00000004568 | ENSGALG00000002869 | ENPP3 | 3 | 59301222 |
| ENSGALT00000031715 | ENSGALG00000019937 | - | 3 | 67197022 |
| ENSGALT00000025514 | - | Q6T5C1 | 3 | 79294329 |
| ENSGALT00000026294 | ENSGALG00000016299 | FAM83B | 3 | 90550564 |
| ENSGALT00000026567 | - | Q684L7 | 3 | 103126324 |
| NM_001168709 | ENSGALG00000016473 | OXSR1 | 3 | 103929131 |
| ENSGALT00000017778 | ENSGALG00000010929 | SPARCL1 | 4 | 47134084 |
| ENSGALT00000037112 | ENSGALG00000019026 | OXT | 4 | 92051627 |
| 603595873F1 | - | - | 5 | 19138531 |
| NM_204768 | ENSGALG00000010213 | VSX2 | 5 | 40231660 |
| NM_001001752 | ENSGALG00000005469 | CYP2C45 | 6 | 17648418 |
| NM_001001752 | ENSGALG00000005469 | CYP2C45 | 6 | 17658058 |
| ENSGALT00000010126 | - | Q6T7C0 | 6 | 20120158 |
| 603866490F1 | - | - | 7 | 11850006 |
| ENSGALT00000033404 | ENSGALG00000020836 | LOC424098 | 7 | 13504156 |
| ENSGALT00000042331 | ENSGALG00000025418 | SNORA41 | 7 | 13642211 |
| NM_205260 | - | GCG | 7 | 22692908 |
| ENSGALT00000019794 | - | EN1 | 7 | 30116760 |
| NM_206989 | ENSGALG00000007222 | UTS2B | 9 | 14792255 |
| ENSGALT00000038746 | ENSGALG00000023436 | HDC | 10 | 12565547 |
| NM_204209 | ENSGALG00000026736 | OGN | 12 | 3642003 |
| 603598354F1 | - | - | 13 | 7238952 |
| 603866983F1 | - | - | 13 | 7239011 |
| ENSGALT00000003729 | ENSGALG00000002367 | LOC417537 | 19 | 4813712 |
| NM_213572 | ENSGALG00000004246 | SLC6A4 | 19 | 6155492 |
| 603862137F1 | - | - | 21 | 1409372 |
| ENSGALT00000000433 | ENSGALG00000000327 | FBN3 | 28 | 246402 |
| ENSGALT00000041334 | - | Q860H5 | 16_random | 170473 |
| 603603069F1 | - | - | Un_random | 17631570 |
| 603862842F1 | - | - | Un_random | 35571649 |
| ENSGALT00000022414 | ENSGALG00000005785 | Q6Y2W3 | W | 13465 |
| ENSGALT00000023597 | ENSGALG00000014641 | - | W_random | 144388 |
| ENSGALT00000021743 | ENSGALG00000013312 | A7XMV1 | W_random | 338407 |
| 603864287F1 | - | - | W_random | 453643 |
| NM_001031404 | ENSGALG00000002419 | KIAA1328 | Z | 6623963 |
| ENSGALT00000009323 | ENSGALG00000005806 | KIF24 | Z | 7097470 |
| ENSGALT00000034419 | - | TLN1 | Z | 8512658 |
| NM_001195689 | ENSGALG00000002599 | C9orf100 | Z | 8622959 |
| ENSGALT00000035392 | ENSGALG00000021847 | - | Z | 9517275 |
| ENSGALT00000039152 | ENSGALG00000023552 | - | Z | 10157826 |
| ENSGALT00000041534 | ENSGALG00000024621 | SNORD72 | Z | 12332205 |
| ENSGALT00000032852 | ENSGALG00000020561 | LOC425372 | Z | 15922066 |
| ENSGALT00000023780 | ENSGALG00000014747 | SDCCAG10 | Z | 19606970 |
| ENSGALT00000038670 | ENSGALG00000023411 | CD180 | Z | 20724015 |
| 603577273F1 | - | - | Z | 20854055 |
| 603597693F1 | - | - | Z | 21550388 |
| ENSGALT00000023909 | - | CMYA5 | Z | 21578618 |
| ENSGALT00000024182 | ENSGALG00000014994 | CRHBP | Z | 22828707 |
| ENSGALT00000024125 | ENSGALG00000014958 | ANKDD1B | Z | 23338056 |
| ENSGALT00000029057 | ENSGALG00000018350 | gga-mir-101 | Z | 28037874 |
| 603865010F1 | - | - | Z | 30610014 |
| 603863044F1 | - | - | Z | 30614314 |
| ENSGALT00000008716 | ENSGALG00000005426 | FREM1 | Z | 31473831 |
| 603602069F1 | - | - | Z | 31684424 |
| ENSGALT00000032827 | ENSGALG00000015097 | CNTLN | Z | 32677922 |
| ENSGALT00000038227 | ENSGALG00000023267 | - | Z | 34252945 |
| 603143690F1 | - | - | Z | 34429048 |
| NM_001006580 | ENSGALG00000015145 | TMC1 | Z | 35506626 |
| 603602242F1 | - | - | Z | 38048676 |
| ENSGALT00000020555 | ENSGALG00000012588 | KIF27 | Z | 39541726 |
| ENSGALT00000020595 | ENSGALG00000012615 | Q5ZK89 | Z | 40977919 |
| NM_001031429 | ENSGALG00000021843 | AUH | Z | 43588754 |
| 603596301F1 | - | - | Z | 44153399 |
| ENSGALT00000037912 | - | CHSY3 | Z | 44286817 |
| ENSGALT00000000240 | ENSGALG00000000184 | SLC27A6 | Z | 44827552 |
| ENSGALT00000028609 | ENSGALG00000017902 | U2 | Z | 44855120 |
| NM_001044634 | ENSGALG00000000247 | WDR36 | Z | 45932568 |
| NM_001097534 | ENSGALG00000015372 | ATP5I | Z | 52365085 |
| 603596432F1 | - | - | Z | 55200083 |
| ENSGALT00000037708 | - | Q9PUC7 | Z | 55839268 |
| ENSGALT00000023643 | ENSGALG00000014672 | ARSK | Z | 56315471 |
| ENSGALT00000023622 | ENSGALG00000014657 | GPR98 | Z | 58491012 |
| ENSGALT00000025179 | ENSGALG00000015620 | XRCC4 | Z | 61535260 |
| 603863839F1 | - | - | Z | 62365197 |
| ENSGALT00000025175 | ENSGALG00000015616 | ACOT12 | Z | 62385185 |
| NM_205230 | ENSGALG00000015691 | SMC2 | Z | 65036306 |
| ENSGALT00000025350 | ENSGALG00000015721 | SVEP1 | Z | 65202109 |
| ENSGALT00000034649 | ENSGALG00000021457 | LOC769382 | Z | 66488462 |
| NM_205202 | ENSGALG00000002022 | B4GALT1 | Z | 68722880 |
| ENSGALT00000003296 | ENSGALG00000002110 | TSTD2 | Z | 68847231 |
| ENSGALT00000003435 | - | TFIP8 | Z | 69693606 |
| 603864076F1 | - | - | Z | 74273781 |
| 603600572F1 | - | - | - | - |
