# Supplementary material for: Long-Term and Transgenerational Effects of Stress Experienced during Different Life Phases in Chickens (Gallus gallus)
Source: PLoS One. 2016 Apr 22;11(4):e0153879. doi: 10.1371/journal.pone.0153879 (PMC4841578; doi:10.1371/journal.pone.0153879)
Supplement: S4 Table — (DOCX) [file pone.0153879.s004.docx]

**Supplementary table 4**

**S4. Differentially expressed probes for each treatment group when comparing parents with their offspring.** Table provides probe transcript IDs as well as Ensembl Gene ID and annotated genes when available. Chromosomal starting point of each probe is given. Logarithmic fold change (logFC) is given for each probe in parents and offspring.

| Treatment | Probe ID | Ensembl Gene ID | Gene | Chromosome | Start | Parents logFC | Offspring logFC |
| --- | --- | --- | --- | --- | --- | --- | --- |
| 2w | NM_001130741 | ENSGALG00000006833 | ITIH2 | 1 | 4126596 | 1,3800 | -1,1051 |
| 2w | ENSGALT00000038498 | ENSGALG00000010198 | Q6PKI7 | 1 | 38382479 | -2,7795 | -2,8963 |
| 2w | NM_001001301 | ENSGALG00000010198 | TPH2 | 1 | 38382634 | -2,6022 | -3,0255 |
| 2w | ENSGALT00000036727 | ENSGALG00000016132 | IGSF5 | 1 | 111556541 | 1,1184 | 0,6296 |
| 2w | ENSGALT00000030284 | - | C4PCM1 | 1 | 126830698 | 0,9746 | -0,8030 |
| 2w | ENSGALT00000027110 | ENSGALG00000016791 | SLC9A2 | 1 | 138232028 | 1,0040 | -1,1295 |
| 2w | ENSGALT00000027127 | ENSGALG00000016804 | SLC5A7 | 1 | 140322894 | -0,8430 | -1,1034 |
| 2w | ENSGALT00000012288 | - | Q98TV1 | 1 | 146892812 | -0,8297 | -0,6498 |
| 2w | 603865102F1 | - | - | 1 | 151820688 | 0,7888 | -0,7000 |
| 2w | NM_001030541 | ENSGALG00000017046 | POSTN | 1 | 176288240 | 1,1546 | -0,8553 |
| 2w | ENSGALT00000027540 | ENSGALG00000017046 | Q6DMS3 | 1 | 176288241 | 1,2563 | -0,9960 |
| 2w | ENSGALT00000036466 | - | Q6DMS3 | 1 | 176288241 | 1,2804 | -0,9215 |
| 2w | ENSGALT00000038721 | ENSGALG00000023430 | EN2 | 2 | 7817560 | -0,8839 | -0,9968 |
| 2w | ENSGALT00000015507 | - | Q700F0 | 2 | 23530917 | 1,0276 | 0,6761 |
| 2w | 603568022F1 | - | - | 2 | 31733237 | 3,7719 | -0,6531 |
| 2w | ENSGALT00000025563 | ENSGALG00000015857 | CA3 | 2 | 127529336 | 0,7664 | 0,6925 |
| 2w | NM_205268 | ENSGALG00000016112 | NOV | 2 | 142002873 | 1,2352 | -0,9673 |
| 2w | ENSGALT00000025959 | ENSGALG00000016112 | NOV | 2 | 142002898 | 1,2645 | -0,9686 |
| 2w | 603568049F1 | - | - | 3 | 18282660 | 1,0235 | 0,7295 |
| 2w | 603867870F1 | - | - | 3 | 56444558 | 1,4811 | -0,7601 |
| 2w | NM_001145990 | ENSGALG00000014917 | VGLL2 | 3 | 66207330 | 0,9387 | 0,7084 |
| 2w | NM_001168709 | ENSGALG00000016473 | OXSR1 | 3 | 103929131 | 0,8842 | -0,7892 |
| 2w | ENSGALT00000041927 | ENSGALG00000025014 | SNORD61 | 4 | 4430956 | 1,2560 | -0,7172 |
| 2w | ENSGALT00000016833 | - | - | 4 | 36154183 | 0,8421 | -0,7548 |
| 2w | ENSGALT00000017775 | ENSGALG00000010926 | SPP1 | 4 | 47110531 | -0,9370 | -0,8430 |
| 2w | ENSGALT00000017778 | ENSGALG00000010929 | SPARCL1 | 4 | 47134084 | -1,2172 | -0,7956 |
| 2w | 603863304F1 | - | - | 4 | 47390635 | -1,0099 | 0,6472 |
| 2w | ENSGALT00000022171 | ENSGALG00000013627 | CTR2 | 4 | 64887385 | 1,3020 | 1,2514 |
| 2w | ENSGALT00000022839 | ENSGALG00000014108 | SLC10A4 | 4 | 68330346 | -2,1228 | -1,6566 |
| 2w | NM_001081501 | ENSGALG00000014363 | CCKAR | 4 | 75629863 | -1,2260 | -1,1688 |
| 2w | ENSGALT00000037112 | ENSGALG00000019026 | OXT | 4 | 92051627 | 1,4886 | -0,6297 |
| 2w | ENSGALT00000030013 | - | - | 4 | 92051627 | 1,4906 | -0,6449 |
| 2w | 603595873F1 | - | - | 5 | 19138531 | 2,1534 | 1,0799 |
| 2w | 603597179F1 | - | - | 5 | 38617877 | -0,8066 | -0,6846 |
| 2w | NM_204768 | ENSGALG00000010213 | VSX2 | 5 | 40231660 | -1,4161 | -2,2378 |
| 2w | ENSGALT00000018666 | ENSGALG00000011446 | TNFAIP2 | 5 | 52656113 | 0,8274 | -0,6621 |
| 2w | 603862655F1 | - | - | 6 | 3930308 | -1,7675 | -0,8132 |
| 2w | NM_204610 | ENSGALG00000002289 | CHAT | 6 | 3964302 | -1,6167 | -0,9235 |
| 2w | NM_001001752 | ENSGALG00000005469 | CYP2C45 | 6 | 17648418 | 1,1292 | 0,6826 |
| 2w | ENSGALT00000008786 | ENSGALG00000005469 | - | 6 | 17648435 | 1,0498 | 0,8671 |
| 2w | NM_001001752 | ENSGALG00000005469 | CYP2C45 | 6 | 17658058 | 1,1292 | 0,6826 |
| 2w | ENSGALT00000040129 | - | Q9PS87 | 6 | 18541789 | -1,4486 | -1,2136 |
| 2w | ENSGALT00000042331 | ENSGALG00000025418 | SNORA41 | 7 | 13642211 | 0,8826 | 0,7298 |
| 2w | NM_001190165 | ENSGALG00000011104 | GCG | 7 | 22692908 | 0,8261 | -0,7835 |
| 2w | ENSGALT00000019794 | - | EN1 | 7 | 30116760 | -0,9424 | -1,8426 |
| 2w | ENSGALT00000039014 | - | Q7T191 | 8 | 14354115 | 1,9111 | 1,2622 |
| 2w | 603864238F1 | - | - | 9 | 2697668 | 1,1788 | 0,9239 |
| 2w | NM_204415 | ENSGALG00000003021 | CHRNA5 | 10 | 4583033 | 0,8023 | -0,8823 |
| 2w | NM_204669 | ENSGALG00000007129 | ALDH1A3 | 10 | 19570925 | 0,9655 | 0,6575 |
| 2w | ENSGALT00000008222 | ENSGALG00000005120 | FBLN2 | 12 | 6019276 | 0,8224 | -0,6501 |
| 2w | NM_001030383 | ENSGALG00000008490 | TRH | 12 | 20356532 | 0,7934 | -0,7584 |
| 2w | ENSGALT00000039311 | - | LOC770114 | 14 | 14403164 | -0,7937 | -0,7948 |
| 2w | ENSGALT00000004933 | ENSGALG00000003120 | NCOR2 | 15 | 4748839 | -0,8704 | -0,7540 |
| 2w | ENSGALT00000039150 | - | Q9I9A1 | 17 | 7683358 | -1,1778 | -2,1603 |
| 2w | NM_001030335 | ENSGALG00000003928 | LHX3 | 17 | 8735541 | 1,1031 | 1,1775 |
| 2w | NM_001177738 | ENSGALG00000023354 | FSCN2 | 18 | 9206838 | -0,8970 | 0,8498 |
| 2w | ENSGALT00000003729 | ENSGALG00000002367 | LOC417537 | 19 | 4813712 | 1,1641 | -0,6311 |
| 2w | NM_213572 | ENSGALG00000004246 | SLC6A4 | 19 | 6155492 | -2,6591 | -2,7173 |
| 2w | ENSGALT00000000755 | ENSGALG00000000543 | TNFRSF9 | 21 | 243622 | -1,0973 | -0,6993 |
| 2w | NM_206990 | ENSGALG00000000556 | UTS2 | 21 | 256326 | -1,0258 | -0,7752 |
| 2w | NM_204121 | ENSGALG00000025817 | NHLH1 | 25 | 1347055 | -1,1898 | -0,8686 |
| 2w | ENSGALT00000018722 | ENSGALG00000011485 | Q8AV17 | 27 | 4508326 | 1,2476 | 0,7368 |
| 2w | 603863336F1 | - | - | 28 | 2335850 | -0,8283 | -0,7807 |
| 2w | ENSGALT00000029097 | ENSGALG00000018377 | - | MT | 6430 | -1,0272 | 1,2754 |
| 2w | 603603069F1 | - | - | Un_random | 17631570 | -2,9533 | -1,1112 |
| 2w | 603862842F1 | - | - | Un_random | 35571649 | 1,2266 | 1,0069 |
| 2w | ENSGALT00000023597 | - | - | W_random | 144388 | -2,8376 | -0,6595 |
| 2w | 603867221F1 | - | - | Z | 1129467 | -1,4491 | -0,9491 |
| 2w | NM_001031404 | ENSGALG00000002419 | LOC426890 | Z | 6623963 | 1,3850 | 0,6923 |
| 2w | ENSGALT00000003394 | ENSGALG00000002165 | UNC13B | Z | 8057456 | 0,9347 | 0,9266 |
| 2w | ENSGALT00000039152 | ENSGALG00000023552 | - | Z | 10157826 | 1,0485 | 0,6799 |
| 2w | NM_204939 | ENSGALG00000014874 | MRPS30 | Z | 13609764 | 0,9985 | 0,7778 |
| 2w | ENSGALT00000038886 | - | FST | Z | 15391867 | 1,4618 | 0,7374 |
| 2w | NM_204457 | ENSGALG00000013548 | GZMA | Z | 15998582 | 0,9029 | -0,9743 |
| 2w | ENSGALT00000038855 | - | - | Z | 16013782 | -0,9730 | 0,6525 |
| 2w | 603865427F1 | - | - | Z | 20818301 | 1,7042 | -1,0866 |
| 2w | 603577273F1 | - | - | Z | 20854055 | 1,2540 | -1,4615 |
| 2w | 603601792F1 | - | - | Z | 21924786 | 1,4681 | 0,6560 |
| 2w | ENSGALT00000000240 | ENSGALG00000000184 | SLC27A6 | Z | 44827552 | 0,9801 | -0,6614 |
| 2w | ENSGALT00000024804 | - | Q802E5 | Z | 52393378 | 2,0745 | -0,6521 |
| 2w | ENSGALT00000037605 | - | SMC2 | Z | 65036283 | 1,4158 | 0,6289 |
| 2w | NM_205230 | ENSGALG00000015691 | SMC2 | Z | 65036306 | 1,2397 | 0,7645 |
| 2w | 603143017F1 | - | - | - | - | - | - |
| 2w | 603143451F1 | - | - | - | - | - | - |
| 2w | 603597107F1 | - | - | - | - | - | - |
| 2w | 603597572F1 | - | - | - | - | - | - |
| 2w | 603598212F1 | - | - | - | - | - | - |
| 2w | 603598755F1 | - | - | - | - | - | - |
| 2w | 603599936F1 | - | - | - | - | - | - |
| 2w | 603601601F1 | - | - | - | - | - | - |
| 2w | 603866318F1 | - | - | - | - | - | - |
| 2w | 603866946F1 | - | - | - | - | - | - |
| 2w | 603867949F1 | - | - | - | - | - | - |
| 2w | 603868105F1 | - | - | - | - | - | - |
| 2w | 603868386F1 | - | - | - | - | - | - |
|  |  |  |  |  |  |  |  |
| 8w | NM_001031332 | ENSGALG00000004897 | GTSE1 | 1 | 16895123 | 0,9609 | 0,9098 |
| 8w | ENSGALT00000013934 | ENSGALG00000008554 | IL17REL | 1 | 21437388 | 1,1107 | 0,9632 |
| 8w | NM_001031479 | ENSGALG00000009497 | AVPR2 | 1 | 30283773 | -1,0330 | 0,8034 |
| 8w | 603143987F1 | - | - | 1 | 35765007 | 1,0628 | -0,7407 |
| 8w | 603868271F1 | - | - | 1 | 75806867 | -1,4841 | 0,8448 |
| 8w | ENSGALT00000037054 | ENSGALG00000014754 | - | 1 | 81218286 | -1,0994 | -1,0730 |
| 8w | ENSGALT00000036952 | ENSGALG00000015018 | CASQ2 | 1 | 83709790 | 1,1982 | -0,6639 |
| 8w | ENSGALT00000024221 | - | CASQ2 | 1 | 83709790 | 1,2036 | -0,7590 |
| 8w | NM_204526 | ENSGALG00000015018 | CASQ2 | 1 | 83775772 | 1,1261 | -0,5931 |
| 8w | ENSGALT00000026387 | ENSGALG00000016357 | PRDX4 | 1 | 121813353 | 0,9471 | 0,7734 |
| 8w | ENSGALT00000030302 | ENSGALG00000019157 | SMPX | 1 | 122571767 | 1,2555 | 0,7545 |
| 8w | NM_001024591 | ENSGALG00000016785 | IL1RL1 | 1 | 138087257 | 0,9892 | 0,7388 |
| 8w | ENSGALT00000027127 | ENSGALG00000016804 | SLC5A7 | 1 | 140322894 | -0,9226 | 0,8234 |
| 8w | ENSGALT00000023466 | ENSGALG00000014545 | LOC427010 | 1 | 165980409 | -1,6740 | 0,8448 |
| 8w | NM_001030541 | ENSGALG00000017046 | POSTN | 1 | 176288240 | 1,6549 | 0,5917 |
| 8w | NM_001081704 | ENSGALG00000017347 | HBE | 1 | 199444834 | -1,1357 | -0,6033 |
| 8w | ENSGALT00000015507 | - | Q700F0 | 2 | 23530917 | 1,8741 | 0,6582 |
| 8w | NM_204739 | - | TWIST1 | 2 | 29583668 | 0,9046 | -1,0771 |
| 8w | 603568022F1 | - | - | 2 | 31733237 | 2,3977 | -1,8837 |
| 8w | NM_205094 | ENSGALG00000012775 | TFAP2A | 2 | 64442589 | -0,9958 | 0,7454 |
| 8w | ENSGALT00000020844 | ENSGALG00000012775 | O13111 | 2 | 64442683 | -1,1215 | 0,8180 |
| 8w | NM_204299 | ENSGALG00000012830 | IRF4 | 2 | 67667007 | -1,3622 | -1,2564 |
| 8w | 603141888F1 | - | - | 2 | 109302304 | -0,8718 | -1,6207 |
| 8w | 603143717F1 | - | - | 2 | 109306422 | -0,9346 | -1,5371 |
| 8w | 603602539F1 | - | - | 2 | 109308582 | -0,8869 | -1,5833 |
| 8w | NM_204645 | ENSGALG00000009690 | CENPF | 3 | 22195531 | 0,9081 | 0,7960 |
| 8w | ENSGALT00000017890 | ENSGALG00000010982 | Q9I9K4 | 3 | 39401227 | 0,8915 | 0,6396 |
| 8w | 603865210F1 | - | - | 3 | 44254920 | 1,0262 | -0,6337 |
| 8w | ENSGALT00000004568 | ENSGALG00000002869 | ENPP3 | 3 | 59301222 | 1,4124 | 0,7628 |
| 8w | ENSGALT00000037424 | - | SAMD3 | 3 | 59968470 | 1,2067 | -0,7046 |
| 8w | 603866017F1 | - | - | 3 | 77313749 | -1,6368 | 1,2228 |
| 8w | ENSGALT00000026525 | ENSGALG00000016442 | RRM2 | 3 | 99396972 | 0,9654 | 0,7619 |
| 8w | ENSGALT00000026567 | - | Q684L7 | 3 | 103126324 | 1,0695 | 0,6365 |
| 8w | 603602457F1 | - | - | 3 | 104831830 | 0,8914 | 0,6409 |
| 8w | NM_204895 | ENSGALG00000016680 | TFAP2B | 3 | 111052692 | -0,9470 | 0,8458 |
| 8w | NM_204464 | ENSGALG00000016684 | RHAG | 3 | 111573998 | -1,7305 | -0,6641 |
| 8w | ENSGALT00000040126 | - | - | 4 | 2516714 | 1,1418 | -1,1733 |
| 8w | ENSGALT00000009384 | ENSGALG00000005842 | GDPD2 | 4 | 2525020 | 1,5212 | -1,1476 |
| 8w | NM_204263 | ENSGALG00000011551 | IGJ | 4 | 51513039 | -2,1474 | -2,0665 |
| 8w | ENSGALT00000009761 | ENSGALG00000006054 | CALCA | 5 | 11507155 | -1,9467 | 1,1879 |
| 8w | ENSGALT00000009762 | - | CALC | 5 | 11507155 | -0,9404 | 1,0141 |
| 8w | ENSGALT00000039600 | - | CALC | 5 | 11507155 | -1,7912 | 1,2638 |
| 8w | 603595873F1 | - | - | 5 | 19138531 | 1,2415 | 1,7129 |
| 8w | 603601624F1 | - | - | 5 | 36619748 | -1,0780 | -0,5913 |
| 8w | 603867188F1 | - | - | 5 | 36621114 | -1,2193 | -0,6867 |
| 8w | ENSGALT00000032472 | - | SIX6 | 5 | 56992344 | 1,5782 | -0,8857 |
| 8w | ENSGALT00000032441 | - | TXNDC1 | 5 | 60605530 | -0,8946 | 0,5944 |
| 8w | 603862655F1 | - | - | 6 | 3930308 | -2,5264 | 1,1587 |
| 8w | ENSGALT00000004758 | ENSGALG00000003013 | C10orf107 | 6 | 9585091 | 0,8999 | -0,5872 |
| 8w | ENSGALT00000009008 | ENSGALG00000005613 | PITX3 | 6 | 17907569 | -1,3035 | 0,6541 |
| 8w | ENSGALT00000015072 | ENSGALG00000009261 | VAX1 | 6 | 30563592 | 1,5457 | -0,8253 |
| 8w | NM_204799 | ENSGALG00000009261 | VAX1 | 6 | 30563628 | 1,4782 | -0,6285 |
| 8w | ENSGALT00000016996 | - | - | 6 | 35367911 | 1,1948 | 0,7227 |
| 8w | 603866490F1 | - | - | 7 | 11850006 | 1,5785 | -0,7391 |
| 8w | ENSGALT00000033404 | ENSGALG00000020836 | LOC424098 | 7 | 13504156 | 1,2645 | 0,6229 |
| 8w | ENSGALT00000014100 | ENSGALG00000008656 | ICOS | 7 | 14464698 | -0,8887 | -0,7065 |
| 8w | NM_001100288 | ENSGALG00000008656 | ICOS | 7 | 14471438 | -1,3796 | -0,7925 |
| 8w | ENSGALT00000014484 | ENSGALG00000008908 | NDF1 | 7 | 15350165 | 0,9431 | -0,8849 |
| 8w | ENSGALT00000017711 | ENSGALG00000010891 | ABCB11 | 7 | 20336455 | -1,0842 | -0,7301 |
| 8w | ENSGALT00000032889 | - | - | 7 | 37136328 | -0,9650 | 0,7071 |
| 8w | ENSGALT00000014698 | ENSGALG00000009032 | PIF1 | 8 | 20316628 | 0,9114 | 0,6584 |
| 8w | ENSGALT00000017363 | ENSGALG00000010673 | SLC1A7 | 8 | 25379533 | 1,1042 | -0,8466 |
| 8w | ENSGALT00000011689 | ENSGALG00000007222 | Q6Q273 | 9 | 14792231 | -1,6754 | 0,6542 |
| 8w | NM_206989 | ENSGALG00000007222 | UTS2D | 9 | 14792255 | -1,7007 | 0,6380 |
| 8w | ENSGALT00000038746 | ENSGALG00000023436 | HDC | 10 | 12565547 | 0,9173 | 1,1846 |
| 8w | 603600344F1 | - | - | 10 | 13794863 | -1,2229 | 2,8670 |
| 8w | ENSGALT00000010881 | ENSGALG00000006725 | PGCA | 10 | 14760022 | 0,8662 | 1,1427 |
| 8w | ENSGALT00000013266 | ENSGALG00000008164 | ADAMTS7 | 10 | 21849704 | 1,0774 | 0,6938 |
| 8w | ENSGALT00000008677 | ENSGALG00000005402 | DYNLRB2 | 11 | 16668352 | -0,8783 | 0,8427 |
| 8w | ENSGALT00000011634 | ENSGALG00000007184 | FEZF2 | 12 | 13422283 | 1,5988 | -0,5806 |
| 8w | ENSGALT00000012647 | - | Q5ZLX1 | 12 | 17104715 | -0,8706 | -0,7527 |
| 8w | ENSGALT00000006879 | ENSGALG00000004320 | FAT2 | 13 | 13035557 | 1,7232 | -1,6615 |
| 8w | NM_001030639 | ENSGALG00000006110 | PLK1 | 14 | 6936893 | 1,1294 | 0,7532 |
| 8w | ENSGALT00000012068 | - | HBAD | 14 | 12726667 | -1,8129 | -0,7939 |
| 8w | ENSGALT00000039311 | - | LOC770114 | 14 | 14403164 | -1,1926 | 0,9059 |
| 8w | ENSGALT00000004933 | ENSGALG00000003120 | NCOR2 | 15 | 4748839 | 1,3814 | -0,6917 |
| 8w | ENSGALT00000038524 | - | - | 15 | 8179220 | -2,5580 | -2,9146 |
| 8w | ENSGALT00000009555 | ENSGALG00000005937 | - | 15 | 8181841 | -3,2564 | -2,7636 |
| 8w | ENSGALT00000034031 | ENSGALG00000005919 | - | 15 | 8184155 | -3,0038 | -2,7811 |
| 8w | ENSGALT00000009564 | ENSGALG00000005946 | - | 15 | 8184817 | -2,9847 | -2,8963 |
| 8w | ENSGALT00000009520 | - | - | 15 | 8189562 | -2,7836 | -2,6906 |
| 8w | ENSGALT00000009519 | ENSGALG00000021139 | LAC | 15 | 8189728 | -2,5397 | -2,3056 |
| 8w | ENSGALT00000009518 | - | LAC | 15 | 8189728 | -2,5237 | -2,3563 |
| 8w | ENSGALT00000038525 | - | LAC | 15 | 8189728 | -2,4221 | -2,3585 |
| 8w | ENSGALT00000034024 | - | - | 15 | 8190795 | -1,9797 | -2,1182 |
| 8w | ENSGALT00000034032 | ENSGALG00000021142 | - | 15 | 8194097 | -2,9620 | -2,0943 |
| 8w | ENSGALT00000009559 | ENSGALG00000021142 | - | 15 | 8194097 | -2,8888 | -1,5271 |
| 8w | ENSGALT00000001978 |  | LOC769366 | 16 | 322516 | -0,9401 | -1,0953 |
| 8w | ENSGALT00000004618 | ENSGALG00000002926 | DBH | 17 | 7683358 | 0,9827 | -1,4089 |
| 8w | ENSGALT00000003729 | ENSGALG00000002367 | LOC417537 | 19 | 4813712 | 1,5294 | 0,6490 |
| 8w | ENSGALT00000006754 | - | Q6PWI2 | 19 | 6155492 | -2,2845 | -0,6354 |
| 8w | 603867059F1 | - | - | 20 | 1034683 | 1,1226 | -0,9189 |
| 8w | 603862477F1 | - | - | 21 | 1828243 | 0,9860 | -1,0465 |
| 8w | ENSGALT00000013968 | ENSGALG00000003782 | PAX7 | 21 | 4432735 | -1,2507 | 0,7519 |
| 8w | NM_204175 | ENSGALG00000006809 | POU2AF1 | 24 | 4356189 | -1,1315 | -0,7064 |
| 8w | ENSGALT00000021640 | ENSGALG00000019019 | NTRK1 | 25 | 832763 | 1,1061 | -0,8363 |
| 8w | NM_204967 | ENSGALG00000003546 | FMOD | 26 | 4952662 | 0,8713 | 0,7334 |
| 8w | ENSGALT00000000433 | ENSGALG00000000327 | FBN3 | 28 | 246402 | 1,8347 | 0,6833 |
| 8w | ENSGALT00000000445 | - | - | 28 | 370031 | 1,3259 | 0,6060 |
| 8w | ENSGALT00000000460 | ENSGALG00000029093 | MR1 | 16_random | 25465 | -1,1616 | -0,8766 |
| 8w | 603601828F1 | - | - | 20_random | 44041 | -1,9413 | 1,4430 |
| 8w | NM_001031332 | ENSGALG00000004897 | - | Un_random | 5614598 | 0,9609 | 0,9098 |
| 8w | 603603069F1 | - | - | Un_random | 17631570 | -2,6854 | 0,6646 |
| 8w | ENSGALT00000022414 | ENSGALG00000005785 | Q6Y2W3 | W | 13465 | -1,8219 | 0,8206 |
| 8w | ENSGALT00000021743 | ENSGALG00000013312 | LOC427025 | W_random | 338407 | -1,8108 | 1,1742 |
| 8w | 603864287F1 | - | - | W_random | 453643 | -2,8297 | 1,2375 |
| 8w | ENSGALT00000023322 | ENSGALG00000014441 | LOC431003 | W_random | 468901 | -1,3515 | 0,6007 |
| 8w | ENSGALT00000039654 | ENSGALG00000023718 | KIAA0427 | Z | 1211388 | 1,0492 | -0,5861 |
| 8w | ENSGALT00000035392 | - | - | Z | 9517275 | 1,8661 | 0,9317 |
| 8w | ENSGALT00000039203 | - | - | Z | 9524519 | 1,6469 | 0,6343 |
| 8w | ENSGALT00000005219 | ENSGALG00000003295 | ADAMTS12 | Z | 9584656 | 1,4521 | 0,8711 |
| 8w | ENSGALT00000005480 | - | - | Z | 10136587 | 2,4585 | 0,5967 |
| 8w | 603865972F1 | - | - | Z | 10188768 | 1,0896 | -0,6193 |
| 8w | 603865427F1 | - | - | Z | 20818301 | 1,8677 | -0,6493 |
| 8w | 603577273F1 | - | - | Z | 20854055 | 1,6133 | -0,8147 |
| 8w | ENSGALT00000038562 | ENSGALG00000023379 | F2RL2 | Z | 22954574 | 0,9667 | 0,6052 |
| 8w | 603597779F1 |  |  | Z | 27457291 | -0,9217 | 0,6802 |
| 8w | 603142342F1 | - | - | - | - | - | - |
| 8w | 603142979F1 | - | - | - | - | - | - |
| 8w | 603143154F1 | - | - | - | - | - | - |
| 8w | 603143216F1 | - | - | - | - | - | - |
| 8w | 603597572F1 | - | - | - | - | - | - |
| 8w | 603597679F1 | - | - | - | - | - | - |
| 8w | 603598212F1 | - | - | - | - | - | - |
| 8w | 603600234F1 | - | - | - | - | - | - |
| 8w | 603600572F1 | - | - | - | - | - | - |
| 8w | 603600994F1 | - | - | - | - | - | - |
| 8w | 603601819F1 | - | - | - | - | - | - |
| 8w | 603602614F1 | - | - | - | - | - | - |
| 8w | 603863110F1 | - | - | - | - | - | - |
| 8w | 603863720F1 | - | - | - | - | - | - |
| 8w | 603864034F1 | - | - | - | - | - | - |
| 8w | 603868105F1 | - | - | - | - | - | - |
|  |  |  |  |  |  |  |  |
| 17w | NM_001130741 | ENSGALG00000006833 | ITIH2 | 1 | 4126596 | 0,9184 | 0,8542 |
| 17w | NM_001031332 | ENSGALG00000004897 | GTSE1 | 1 | 16895123 | 0,7596 | 0,8279 |
| 17w | ENSGALT00000038498 | ENSGALG00000010198 | TPH2 | 1 | 38382479 | -1,6543 | -2,3920 |
| 17w | NM_001001301 | ENSGALG00000010198 | TPH2 | 1 | 38382634 | -1,7314 | -2,3570 |
| 17w | 603601667F1 | - | - | 1 | 47587261 | 1,0407 | 0,7917 |
| 17w | NM_001195795 | ENSGALG00000012757 | PMCH | 1 | 57419554 | 1,0621 | 1,6161 |
| 17w | ENSGALT00000020819 | ENSGALG00000012757 | PMCH | 1 | 57419753 | 0,9045 | 1,5624 |
| 17w | ENSGALT00000030545 | - | - | 1 | 63989377 | 2,1676 | -1,3076 |
| 17w | 603596168F1 | - | - | 1 | 67422621 | 0,8050 | 0,8227 |
| 17w | ENSGALT00000037054 | ENSGALG00000014754 | - | 1 | 81218286 | 0,7829 | -1,1043 |
| 17w | ENSGALT00000025369 | ENSGALG00000015730 | PRSS7 | 1 | 102986518 | 0,8857 | 0,8768 |
| 17w | NM_205343 | ENSGALG00000016685 | ASMT | 1 | 133133572 | 1,4634 | 1,8864 |
| 17w | ENSGALT00000027044 | - | - | 1 | 136111094 | 0,6664 | -0,7991 |
| 17w | 603601429F1 | - | - | 1 | 158951976 | 1,5780 | 0,9222 |
| 17w | ENSGALT00000023466 | ENSGALG00000014545 | LOC427010 | 1 | 165980409 | -0,7851 | 1,5884 |
| 17w | ENSGALT00000030159 | - | LOC418847 | 1 | 172106767 | 0,6661 | 0,9857 |
| 17w | ENSGALT00000027534 | ENSGALG00000017041 | FREM2 | 1 | 175741842 | 0,7296 | 0,8700 |
| 17w | NM_001030541 | ENSGALG00000017046 | POSTN | 1 | 176288240 | 1,0604 | 0,8336 |
| 17w | ENSGALT00000038721 | ENSGALG00000023430 | EN2 | 2 | 7817560 | -2,0647 | -0,9786 |
| 17w | ENSGALT00000037700 | - | EOMES | 2 | 38613136 | 0,7666 | -2,0445 |
| 17w | ENSGALT00000037457 | - | - | 2 | 49208370 | 1,5661 | 1,1031 |
| 17w | 603597482F1 | - | - | 2 | 103288526 | -0,7023 | 1,0279 |
| 17w | ENSGALT00000025950 | - | KCNV1 | 2 | 138092795 | -1,2422 | -1,3930 |
| 17w | 603867870F1 | - | - | 3 | 56444558 | 1,0343 | 1,2375 |
| 17w | ENSGALT00000004568 | ENSGALG00000002869 | ENPP3 | 3 | 59301222 | 0,8192 | 1,0598 |
| 17w | ENSGALT00000028822 | - | - | 3 | 74405911 | 0,7540 | 0,8170 |
| 17w | ENSGALT00000025514 | - | Q6T5C1 | 3 | 79294329 | 1,1167 | -0,9620 |
| 17w | 603599921F1 | - | - | 3 | 83378547 | 0,7061 | 0,9311 |
| 17w | ENSGALT00000026567 | - | Q684L7 | 3 | 103126324 | 0,7576 | 0,9347 |
| 17w | 603596651F1 | - | - | 3 | 107142882 | 0,8808 | 0,8492 |
| 17w | 603866422F1 | - | - | 4 | 8012797 | 0,6696 | 0,8854 |
| 17w | NM_205011 | ENSGALG00000010402 | HPGDS | 4 | 38391036 | -0,7525 | -1,2258 |
| 17w | 603866446F1 | - | - | 4 | 54643994 | -0,6618 | -0,8685 |
| 17w | ENSGALT00000032082 | - | - | 4 | 69019429 | 0,8301 | 0,8062 |
| 17w | 603862771F1 | - | - | 4 | 85990776 | 0,7648 | 0,7875 |
| 17w | ENSGALT00000042518 | ENSGALG00000025605 | SCARNA23 | 4 | 86235369 | 0,7308 | -0,8448 |
| 17w | ENSGALT00000037112 | ENSGALG00000019026 | OXT | 4 | 92051627 | 0,8042 | -1,9985 |
| 17w | ENSGALT00000030013 | - | Q2ACD0 | 4 | 92051627 | 0,7571 | -1,8134 |
| 17w | 603595873F1 | - | - | 5 | 19138531 | 1,1016 | 1,9944 |
| 17w | 603143649F1 | - | - | 5 | 35881447 | 0,6923 | -0,8333 |
| 17w | 603143975F1 | - | - | 5 | 38427182 | 1,4051 | 0,8566 |
| 17w | NM_204768 | ENSGALG00000010213 | VSX2 | 5 | 40231660 | -1,5976 | -1,0683 |
| 17w | NM_001122648 | - | DIO3 | 5 | 51792771 | -0,8178 | -1,0218 |
| 17w | NM_205223 | ENSGALG00000012200 | GCH1 | 5 | 58923104 | -0,6835 | -0,8331 |
| 17w | ENSGALT00000009124 | ENSGALG00000005689 | Q9PTX1 | 6 | 18258588 | -0,9895 | -1,0781 |
| 17w | ENSGALT00000010126 | - | Q6T7C0 | 6 | 20120158 | 0,9839 | 0,8701 |
| 17w | ENSGALT00000015072 | ENSGALG00000009261 | VAX1 | 6 | 30563592 | 0,7680 | -0,8919 |
| 17w | NM_204799 | ENSGALG00000009261 | VAX1 | 6 | 30563628 | 0,7586 | -0,8343 |
| 17w | ENSGALT00000015112 | ENSGALG00000009289 | SLC18A2 | 6 | 30619893 | -1,4154 | -1,3319 |
| 17w | ENSGALT00000042006 | ENSGALG00000025093 | SNORD70 | 7 | 12847697 | 0,7335 | 1,2159 |
| 17w | ENSGALT00000033404 | ENSGALG00000020836 | LOC424098 | 7 | 13504156 | 1,5580 | 1,4605 |
| 17w | ENSGALT00000042331 | ENSGALG00000025418 | SNORA41 | 7 | 13642211 | 0,9782 | 1,0479 |
| 17w | ENSGALT00000017673 | - | RDH5 | 7 | 20326319 | -1,4858 | -0,8336 |
| 17w | ENSGALT00000017711 | ENSGALG00000010891 | ABCB11 | 7 | 20336455 | -0,7892 | -0,9188 |
| 17w | ENSGALT00000018128 | ENSGALG00000011122 | TBR1 | 7 | 22959673 | 0,7318 | -1,2121 |
| 17w | ENSGALT00000019794 | - | EN1 | 7 | 30116760 | -2,1497 | -1,3837 |
| 17w | ENSGALT00000020268 | - | LRP1B | 7 | 33901152 | 0,8015 | 1,7453 |
| 17w | ENSGALT00000039014 | - | Q7T191 | 8 | 14354115 | 1,2385 | 2,7038 |
| 17w | 603864238F1 | - | - | 9 | 2697668 | 0,7838 | 1,0960 |
| 17w | ENSGALT00000011622 | ENSGALG00000007179 | ATP13A5 | 9 | 14223006 | 0,8306 | -1,0989 |
| 17w | 603599863F1 | - | - | 9 | 14249853 | 0,8040 | -1,0946 |
| 17w | NM_206989 | ENSGALG00000007222 | UTS2D | 9 | 14792255 | -0,6761 | -0,8794 |
| 17w | ENSGALT00000038831 | - | - | 10 | 12374764 | -0,8480 | 0,8446 |
| 17w | ENSGALT00000038746 | ENSGALG00000023436 | HDC | 10 | 12565547 | 1,0797 | 1,9151 |
| 17w | ENSGALT00000011024 | ENSGALG00000006819 | AGBL1 | 10 | 15565823 | 0,9883 | 0,8666 |
| 17w | ENSGALT00000036227 | - | - | 12 | 14596997 | 1,0426 | 1,7804 |
| 17w | 603866983F1 | - | - | 13 | 7239011 | -1,7828 | 1,4808 |
| 17w | NM_001144848 | ENSGALG00000003270 | DRD1 | 13 | 10106758 | 0,9863 | 0,9142 |
| 17w | ENSGALT00000034218 | ENSGALG00000021235 | Q2I810 | 13 | 14714859 | 0,9171 | 1,0143 |
| 17w | NM_001044687 | ENSGALG00000021235 | NPY6R | 13 | 14714916 | 0,9728 | 1,0418 |
| 17w | 603865101F1 | - | - | 14 | 7493093 | 0,8363 | 0,8346 |
| 17w | ENSGALT00000034024 | - | - | 15 | 8190795 | -1,0228 | -2,0788 |
| 17w | NM_001044682 | ENSGALG00000000136 | B-NK | 16 | 76418 | -0,8268 | -0,8842 |
| 17w | ENSGALT00000002057 | ENSGALG00000001347 | LHX6 | 17 | 9441862 | 0,6866 | -0,8548 |
| 17w | ENSGALT00000035245 | ENSGALG00000021710 | GSG1L | 18 | 123556 | 0,9092 | -1,8129 |
| 17w | ENSGALT00000003729 | ENSGALG00000002367 | LOC417537 | 19 | 4813712 | 1,1754 | 0,8584 |
| 17w | NM_213572 | ENSGALG00000004246 | SLC6A4 | 19 | 6155492 | -2,1500 | -2,2936 |
| 17w | ENSGALT00000006754 | - | Q6PWI2 | 19 | 6155492 | -2,2775 | -2,7076 |
| 17w | ENSGALT00000008607 | ENSGALG00000005362 | LOC772204 | 19 | 8075818 | 0,6731 | 0,7931 |
| 17w | NM_001012695 | ENSGALG00000001141 | HES5 | 21 | 1408371 | 1,9616 | 0,8896 |
| 17w | 603596133F1 | - | - | 21 | 1826465 | 0,9721 | -0,8830 |
| 17w | ENSGALT00000012457 | ENSGALG00000007685 | BCL9L | 24 | 5735256 | -0,8765 | 1,7617 |
| 17w | 603602543F1 | - | - | 26 | 1625292 | 0,8490 | 0,8215 |
| 17w | NM_001044644 | ENSGALG00000000919 | PIGR | 26 | 2401255 | 0,7231 | 1,7654 |
| 17w | ENSGALT00000039851 | ENSGALG00000023791 | - | 26 | 4496543 | 0,7466 | -1,1290 |
| 17w | ENSGALT00000004107 | ENSGALG00000014600 | Q802S7 | 16_random | 210574 | -0,7784 | -0,8257 |
| 17w | ENSGALT00000021823 | ENSGALG00000013378 | CTSG | 28_random | 91901 | 0,7686 | -0,9627 |
| 17w | NM_001031332 | ENSGALG00000004897 | GTSE1 | Un_random | 5614598 | 0,7596 | 0,8279 |
| 17w | 603603069F1 | - | - | Un_random | 17631570 | -1,1111 | 2,9276 |
| 17w | ENSGALT00000022414 | ENSGALG00000005785 | Q6Y2W3 | W | 13465 | -0,6800 | 1,3578 |
| 17w | ENSGALT00000023597 | - | - | W_random | 144388 | -1,3856 | 1,6970 |
| 17w | ENSGALT00000021743 | ENSGALG00000013312 | A7XMV1 | W_random | 338407 | -0,8888 | 2,3378 |
| 17w | 603864287F1 | - | - | W_random | 453643 | -1,1974 | 2,4128 |
| 17w | 603144452F1 | - | - | Z | 793941 | -0,9927 | -0,8921 |
| 17w | ENSGALT00000003855 | ENSGALG00000023622 | AVID | Z | 8485802 | 0,7561 | -1,0388 |
| 17w | NM_205320 | ENSGALG00000023622 | LOC396260 | Z | 8501238 | 0,7651 | -1,0154 |
| 17w | ENSGALT00000035392 | - | - | Z | 9517275 | 0,8033 | 1,1291 |
| 17w | ENSGALT00000005480 | - | - | Z | 10136587 | 2,0586 | 1,1866 |
| 17w | 603865972F1 | - | - | Z | 10188768 | 1,2388 | -1,2048 |
| 17w | NM_001080106 | ENSGALG00000013372 | IL7R | Z | 10231993 | 0,6799 | -1,0148 |
| 17w | ENSGALT00000038886 | - | FST | Z | 15391867 | 0,7846 | 0,8393 |
| 17w | NM_204457 | ENSGALG00000013548 | GZMA | Z | 15998582 | 1,0009 | -0,9612 |
| 17w | 603595555F1 | - | - | Z | 16818661 | 1,3794 | 1,7779 |
| 17w | 603600893F1 | - | - | Z | 21323778 | 1,2655 | -1,1145 |
| 17w | ENSGALT00000025405 | ENSGALG00000015753 | LOC431640 | Z | 65604622 | 0,9899 | 0,7925 |
| 17w | ENSGALT00000002697 | - | - | Z | 66481030 | 0,7093 | -0,8175 |
| 17w | 603143154F1 | - | - | - | - | - | - |
| 17w | 603596758F1 | - | - | - | - | - | - |
| 17w | 603597721F1 | - | - | - | - | - | - |
| 17w | 603599786F1 | - | - | - | - | - | - |
| 17w | 603603032F1 | - | - | - | - | - | - |
| 17w | 603603249F1 | - | - | - | - | - | - |
| 17w | 603863532F1 | - | - | - | - | - | - |
| 17w | 603868105F1 | - | - | - | - | - | - |
|  |  |  |  |  |  |  |  |
